# Synergistic Enhancement of HCF Lifespan in Carbon–Kevlar/Epoxy Hybrid Composites UsingSilica and Graphene Nanoparticles

**DOI:** 10.3390/polym18070866

**Published:** 2026-04-01

**Authors:** Iman Voghofi, Faramarz Ashenai Ghasemi, Kazem Reza Kashyzadeh

**Affiliations:** 1 Solids Design Group, Mechanical Engineering Faculty, Shahid Rajaee Teacher Training University, Tehran 16788-15811, Iran; 2 Department of Transport Equipment and Technology, Academy of Engineering, RUDN University, 6 Miklukho-Maklaya Street, Moscow 117198, Russia

**Keywords:** polymer matrix composites, multi-scale composites, nanoparticle reinforcement, fatigue behavior, high-cycle fatigue life

## Abstract

High-cycle fatigue (HCF) behavior of multi-scale hybrid composites remains a critical area of investigation for advanced applications in aerospace and automotive industries. This study aims to experimentally investigate and optimize the HCF performance of carbon–Kevlar/epoxy hybrid composites through synergistic incorporation of nano-silica (nSiO_2_) and nano-graphene (nGr). Laminates were fabricated using a hand lay-up process followed by press molding, with a [2 carbon fiber/4 Kevlar fiber/2 carbon fiber] stacking sequence. Sixteen material configurations were investigated based on a Taguchi design of experiment (DOE), with two input parameters (nanoparticle percentages) at four different levels each. Following tensile screening tests, three optimal formulations were selected for fatigue evaluation alongside a non-reinforced baseline. Axial fatigue tests were conducted under load-controlled conditions with a stress ratio of R = 0.01 at a constant frequency of 5 Hz. Stress levels were set at 65%, 70%, and 75% of the ultimate tensile strength (UTS), which ranged from 211 MPa for the baseline composite to 390 MPa for the optimal hybrid formulation (1.2 wt.% nSiO_2_ and 0.75 wt.% nGr). Scanning electron microscopy (SEM) analysis of fracture surfaces was performed to correlate microstructural features with fatigue performance. The results demonstrate a remarkable synergistic effect. The optimal hybrid nanocomposite exhibited superior fatigue life, sustaining significantly higher maximum stress (253 MPa vs. 137 MPa at 65% UTS) and achieving a life increase of several-fold compared to the non-modified baseline. SEM observations revealed that this enhancement stems from complementary microstructural mechanisms: nSiO_2_ particles are uniformly dispersed without agglomeration, providing matrix toughening through crack deflection, while nGr sheets enhance interfacial adhesion, as evidenced by complete matrix coverage on fiber surfaces. The optimal formulation uniquely displays both mechanisms operating simultaneously, creating a true multi-scale reinforcement architecture. In contrast, sub-optimal formulations showed nanoparticle agglomerations that acted as stress concentrators under cyclic loading, explaining their intermediate fatigue performance despite high static strength.

## 1. Introduction

Fiber-reinforced polymer (FRP) composites have become indispensable in the design of modern lightweight structures across the aerospace, automotive, and marine industries, owing to their exceptional specific strength and stiffness, corrosion resistance, and tailorability [1,2]. The pursuit of optimal performance has led to the development of hybrid composites, which combine different types of fibers within a single matrix to achieve a balance of properties unattainable with a single fiber type [3]. Among these, carbon/Kevlar hybrid composites represent a strategic material system, merging the high strength and stiffness of carbon fibers (tensile strength: ~2500–3500 MPa, tensile modulus: ~230–400 GPa) with the superior impact resistance and toughness of Kevlar fibers (tensile strength: ~2800–3000 MPa, elongation at break: 2.8–3.6%) [4,5].

Wang et al. [6] reported that carbon/Kevlar hybrid composites exhibited 102.93% higher tensile strength and 131.65% higher tensile modulus compared to single Kevlar fiber composites, while elongation at break decreased by 76.13%. In comparison, carbon/glass hybrid composites showed intermediate properties (~500 MPa tensile strength) between pure carbon (~550 MPa) and pure glass (~450 MPa) [7]. Abdurohman and Adhitya [8] demonstrated that Kevlar/carbon hybrid composites exhibited superior tensile and compressive strength compared to glass/carbon hybrid composites. SEM micrograph analysis revealed that K/carbon composites have better fiber–matrix adhesion and interlayer bonding than glass/carbon hybrids. Rajamurugan et al. [9] studied Kevlar/glass hybrid composites and found that optimal configurations achieved tensile strengths of ~199 MPa, which is substantially lower than carbon/Kevlar hybrids, highlighting the critical role of carbon fibers in load-bearing capacity. Ismail et al. [10] investigated flax/carbon/Kevlar hybrid composites and found that increasing the carbon/Kevlar content improved tensile modulus by 25.96% and impact strength by 16.05% compared to carbon/Kevlar alone, demonstrating the synergistic potential of multi-fiber hybridization.

The superior performance of carbon/Kevlar hybrids stems from the complementary failure modes of the two fibers: carbon fibers exhibit clean, brittle fractures with neat breaks, while Kevlar fibers undergo irregular fractures with yarn splitting and fiber fibrillation, which absorb significant energy during failure [11]. This brittle–ductile combination creates a hybrid effect where the composite benefits from both high stiffness (from carbon) and enhanced damage tolerance (from Kevlar), making it particularly attractive for applications such as high-speed vessels and protective structures.

A critical factor limiting the service life of such structures is fatigue failure [12,13]. Under cyclic loading, composite materials undergo complex damage mechanisms including matrix cracking, fiber–matrix debonding, delamination, and fiber breakage [14,15,16]. Unlike metals, composites do not exhibit a single dominant crack; instead, damage accumulates diffusively, making fatigue life prediction and enhancement a significant challenge [17,18]. Fatigue behavior is inherently matrix-dominated in off-axis and interlaminar regions, meaning the properties of the epoxy resin and the fiber–matrix interface are paramount [19]. Consequently, strategies to improve the fatigue performance of FRPs often focus on enhancing matrix toughness and the integrity of the interfacial bond.

In recent years, nanomodification of polymer matrices has emerged as a powerful frontier for creating next-generation composites with enhanced mechanical properties [20,21]. By incorporating nanoscale reinforcements, a “multi-scale” composite is formed, which can significantly improve matrix-dominated properties. Two prominent nanoparticles for this purpose are nano-silica (nSiO_2_) and nano-graphene (nGr). Nano-silica particles are known to act as effective toughening agents; their homogeneous dispersion in an epoxy matrix can induce crack pinning, deflection, and plastic void growth, thereby increasing fracture toughness and retarding the initiation and growth of micro-cracks [22,23]. On the other hand, nano-graphene, with its high specific surface area and exceptional mechanical properties, can significantly enhance stiffness, strength, and interfacial adhesion. Two-dimensional graphene sheets can bridge cracks and act as barriers to their propagation, while also improving stress transfer between the fiber and matrix [24,25].

Several studies have independently demonstrated the benefits of these nanoparticles. For instance, Khalili et al. [26] showed that a hybrid of nSiO_2_ and nGr improved the Charpy impact strength of basalt–epoxy composites. Similarly, research on the fatigue life prediction of composites has advanced significantly, employing various micromechanical and macromechanical models [27,28,29,30]. However, a survey of the literature reveals a conspicuous gap: while the individual effects of nSiO_2_ or nGr have been explored, their synergistic potential for enhancing the high-cycle fatigue performance of carbon/Kevlar hybrid composites remains largely unexplored.

Therefore, this study aims to conduct a comprehensive experimental investigation into the high-cycle fatigue behavior of multi-scale carbon–Kevlar/epoxy composites modified with a hybrid system of nano-silica and nano-graphene. The specific objectives are:

1. To fabricate carbon/Kevlar hybrid composites with systematically varied weight percentages of nSiO_2_ and nGr using a Taguchi DOE.
2. To identify optimal formulations through tensile screening of 16 configurations.
3. To characterize the tensile–tensile fatigue life of selected composites under constant amplitude loading (R ≈ 0).
4. To quantify the individual and synergistic effects of the nanoparticles on fatigue performance.
5. To discuss underlying mechanisms responsible for observed fatigue life enhancement.

By systematically addressing these points, this work provides valuable insights and experimental data for developing more durable and reliable hybrid composite materials for demanding engineering applications.

## 2. Materials and Methods

### 2.1. Raw Materials

The materials used in this study consisted of an epoxy matrix, reinforcing fibers, and nanoscale fillers.

- Matrix: A two-part, cold-curing epoxy system (R630 resin and H630 hardener, Composite Kavian Co., Tehran, Iran) was used. These components exhibit suitable viscosities for hand lay-up processing. Detailed physical and mechanical properties are provided in Table 1.

- Reinforcement: Carbon fiber (CF) and Kevlar fiber (KF) were supplied by Sazeh Sanat Research Polymer Co. (Tehran, Iran). Key properties of the fibers, as provided by the manufacturer, are listed in Table 2.

- Nanoparticles: Two types of nanoparticles were used: graphene nano-powder (purity: ~78% Carbon, 4–8 layers, 5–10 µm sheet diameter, Fine-Nano Co., Tehran, Iran) and silica nanoparticles (SiO_2_, purity: 99.98%, particle size: 20–25 nm, Fine-Nano Co., Tehran, Iran) [37]. Additional characterization of these nanoparticles is available in the authors’ previous research work [36].

### 2.2. Composite Fabrication and Specimen Preparation

**Figure 1:**
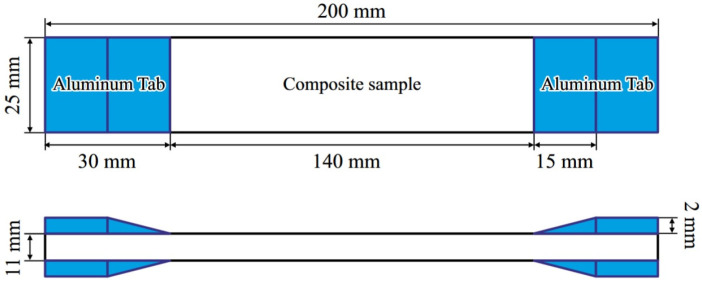
Test specimen geometry with aluminum end tabs. Reprinted from Ref. [39].

Four distinct composite formulations were selected for fatigue testing, as detailed in Table 3. These formulations were identified through an initial screening process using a Taguchi Design of Experiment on 16 different nanoparticle combinations (see Section 3.1). The manufacturing process involved several key steps:

1. Nanoparticle Dispersion: Pre-calculated weights of nSiO_2_ and nGr were added to the R630 epoxy resin. The mixture was mechanically stirred at 2000 rpm for 15 min, followed by ultrasonic treatment for 15 min to ensure homogeneous dispersion and break up agglomerates.
2. Resin Mixing: H630 hardener was added to the nanomodified resin at the specified 100:30 ratios and mixed gently to minimize air entrapment.
3. Hand Lay-up: A symmetric [CF/CF/KF/KF/KF/KF/CF/CF] stacking sequence was used, where ‘CF’ denotes a carbon fiber ply and ‘KF’ denotes a Kevlar fiber ply. The resin mixture was manually applied to each ply within a waxed mold.
4. Press Molding and Curing: The lay-up was consolidated under pressure for 24 h at room temperature. Subsequently, a post-cure cycle was applied (4 h at 100 °C) to achieve full cross-linking and optimal thermal properties.
5. Specimen Machining: Panels were cut into standard fatigue coupons using a waterjet cutter according to ASTM D3039 [38]. Aluminum end tabs were adhesively bonded to both ends to prevent grip-induced failures, as shown in Figure 1.

**Figure 2:**
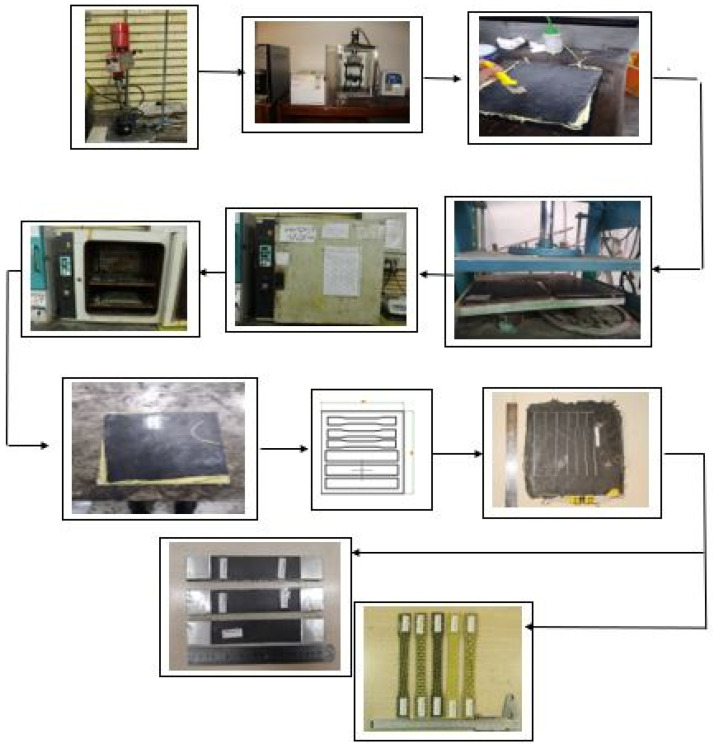
Visual flowchart of the composite sample fabrication method. Adapted from Ref. [36].

A total of 96 samples were prepared. Subsequently, 48 samples were used for tensile testing (3 per case in Taguchi DOE), and 48 samples were used for fatigue testing (12 per formulation). Figure 2 presents a visual flowchart of the composite sample fabrication process.

### 2.3. Mechanical Testing

#### 2.3.1. Tensile Testing

Quasi-static tensile tests were performed on three specimens per formulation using a universal testing machine (SANTAM STM-150, SANTAM Co., Tehran, Iran) equipped with a 150 kN load cell (load capacity: 150 kN, accuracy: ±0.5% of reading value) at a crosshead speed of 2 mm/min, following ASTM D3039 [38]. The results provided the ultimate tensile strength values used to define fatigue stress levels.

#### 2.3.2. Axial Fatigue Testing

High-Cycle Fatigue (HCF) tests were conducted under load-controlled, tension-tension loading (R ≈ 0) using a servo-hydraulic testing machine (SANTAM SAF-50, SANTAM Co., Tehran, Iran). Tests were run at a frequency of 5 Hz and in accordance with ASTM D3479, Standard Test Method for Tension–Tension Fatigue of Polymer Matrix Composite Materials [40]. For each composite formulation, specimens were tested at three stress levels: 65%, 70%, and 75% of their respective UTS. Testing continued until specimen failure or until a pre-defined run-out limit of 1 × 10^6^ cycles was reached. A minimum of four replicates were tested at each condition.

## 3. Results and Discussion

### 3.1. Taguchi Design of Experiment and Tensile Screening

To systematically investigate the effect of nanoparticle concentrations on tensile properties, a Taguchi Design of Experiment (DOE) approach was employed. Two control factors were selected: nano-silica (nSiO_2_) content and nano-graphene (nGr) content, each at four levels, as shown in Table 4.

A Taguchi orthogonal array of L16 (4^2) was employed. This approach was selected to ensure comprehensive coverage of the design space and to systematically manage the different nanoparticle combinations [41,42,43]. For each experimental condition, three replicate specimens were tested (total 48 tensile tests), and the average ultimate tensile strength (UTS) and elastic modulus were recorded as response variables (Table 5).

Based on these results, four formulations were selected for detailed fatigue investigation, considering both ultimate tensile strength and elastic modulus. The selection strategy was as follows:

- C41K41: Baseline (no nanoparticles)—reference for comparison
- C407K407: Highest UTS (390 MPa)—optimal static strength
- C43K43: Highest elastic modulus (41.6 GPa)—optimal stiffness
- C401K401: Balanced (high UTS + high modulus)—hybrid system with good combined properties

Table 3 summarizes the selected formulations. The tensile test results reveal that the addition of nanoparticles significantly increased UTS, with the C407K407 formulation achieving the highest value of 390 MPa, representing an 85% improvement over the unmodified baseline (C41K41). While such improvement may appear substantial, it is consistent with recent literature on hybrid nanoparticle reinforcement. Ai et al. demonstrated 72% and 63% improvements on the impact energy of an epoxy polymer by adding only 2.5% weight of ${Al}_{2}O_{3}$ and $SiO_{2}$ reinforcers, respectively [44]. In another study, it was shown that adding 2% by weight of micron- and nano-sized aluminum particles to a glass fiber-reinforced epoxy polymer composite could increase the ultimate tensile strength of the component by about 20% and 35%, respectively [45].

### 3.2. Fatigue Performance

**Figure 3:**
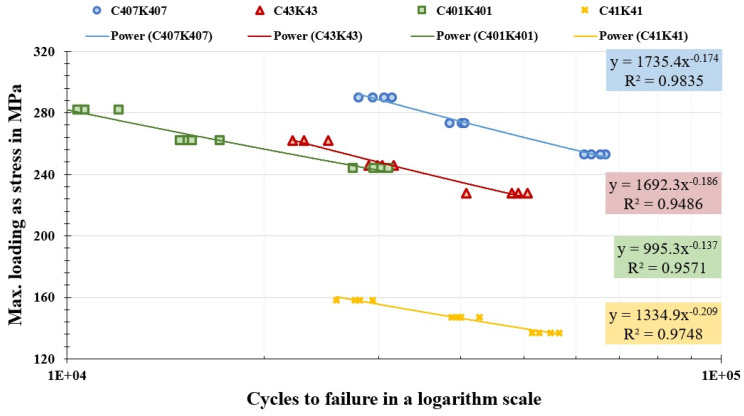
Comparison of high-cycle fatigue behavior for the four selected nanoparticle-reinforced multi-scale composites (S–N curves).

Fatigue test results for four selected formulations are presented as S–N diagrams in Figure 3. Quantitative fatigue data are provided in Appendix A (Table A1). The data unequivocally demonstrates a beneficial effect of nanoparticle addition on fatigue life.

The experimental results reveal a clear hierarchy in fatigue performance: C407K407 > C43K43 > C401K401 > C41K41. This order highlights two critical findings: (1) the addition of any nanoparticle improves fatigue life over baseline, and (2) the hybrid combination of nSiO_2_ and nGr (C407K407) yields a synergistic effect superior to nGr alone (C43K43) or a less-optimized hybrid ratio (C401K401).

The most striking result is the performance of the C407K407 composite at the 65% UTS level. While the baseline (C41K41) failed at approximately 54,000 cycles under 137 MPa, C407K407 sustained a much higher stress of 253 MPa for over 63,000 cycles. This represents not just a life extension, but also an ability to operate at an 85% higher stress level for a comparable or greater number of cycles. This translates to a substantial increase in useful design stress for structural components, which is of paramount engineering significance.

### 3.3. Discussion of Reinforcement Mechanisms

The underlying mechanisms for this enhancement can be understood through the complementary roles of the two nanoparticle types. Well-dispersed silica nanoparticles act as rigid, nano-scale obstacles within the epoxy matrix. During cyclic loading, they effectively pin and deflect micro-cracks, forcing them to follow more tortuous paths. This increases fracture surface area and dissipates more energy, thereby slowing crack growth and delaying failure [22,23].

Additionally, graphene sheets, with their high aspect ratio and surface area, enhance the fiber–matrix interface. They improve stress transfer from the relatively weak matrix to the strong fibers, delaying the onset of debonding—a primary failure initiator in composites under fatigue. Furthermore, graphene sheets can bridge incipient cracks, effectively acting as nano-scale reinforcements that impede crack opening [24,25].

The synergy observed in C407K407 likely stems from a multi-scale toughening mechanism. The nSiO_2_ particles toughen the matrix bulk, making it more resistant to micro-cracking. Concurrently, nGr strengthens the interfaces and bridges micro-cracks that form despite the toughened matrix. This combined action creates a more robust damage-tolerant network throughout the composite structure.

It is noteworthy that the C401K401 formulation (1.0% nSiO_2_, 0.3% nGr) underperformed compared to C43K43 (0.5% nGr only), particularly at higher stress levels (70–75% UTS). This suggests that nanoparticle ratios are crucial. An imbalance or sub-optimal dispersion at this specific ratio may not fully realize the synergistic potential, or it may introduce stress concentrations that become detrimental under higher loads. This highlights the importance of the systematic experimental optimization conducted in this study.

The practical implication of this work is significant. By incorporating a relatively small total weight fraction (~2 wt.%) of inexpensive nanoparticles via a standard manufacturing process, the fatigue lifespan and load-bearing capacity of a carbon/Kevlar hybrid composite can be dramatically enhanced. This offers a cost-effective route to developing more durable composite structures for aerospace, automotive, and marine applications where weight and fatigue resistance are critical design constraints.

The superior performance of carbon/Kevlar hybrids observed in this study is consistent with the broader literature on hybrid composites. Compared to other hybrid systems such as carbon/glass or Kevlar/glass, carbon/Kevlar combinations offer the best balance of stiffness and impact resistance [6,7,8]. The 85% improvement in fatigue stress achieved with optimal nanoparticle reinforcement in this work further enhances this advantage, positioning carbon–Kevlar/nanoparticle multi-scale composites as leading candidates for high-performance structural applications.

### 3.4. Fractographic Analysis Using Scanning Electron Microscopy

**Figure 4:**
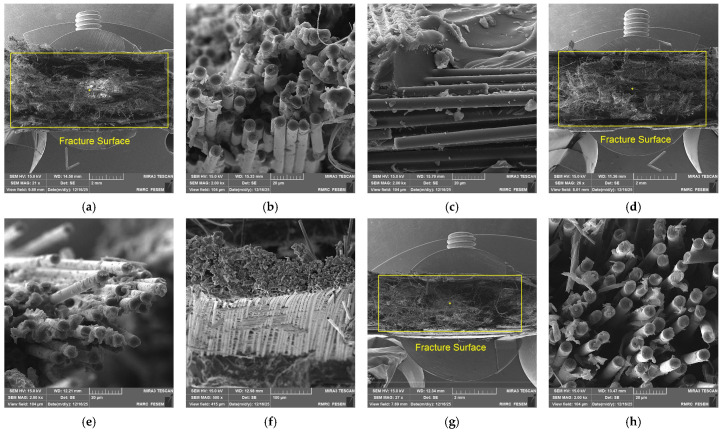

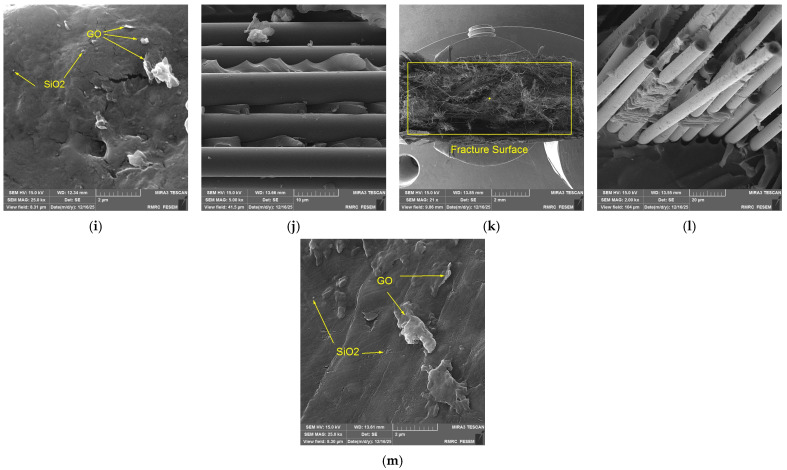
SEM micrographs of fatigue fracture surfaces (65% UTS): (**a**–**c**) C41K41 baseline showing clean fiber pull-out and brittle matrix fracture; (**d**–**f**) C43K43 (0.5% nGr) showing improved fiber–matrix adhesion with matrix remnants on fibers; (**g**–**j**) C401K401 (1.0% SiO_2_ + 0.3% nGr) revealing partial adhesion and nanoparticle agglomerates (circled); (**k**–**m**) C407K407 (1.2% SiO_2_ + 0.75% nGr) displaying complete matrix coverage, exceptional interfacial bonding, and perfectly dispersed nanoparticles (arrows). Magnifications: (**a**,**d**,**g**,**k**) 21–27× overview; (**b**,**e**,**h**,**l**) 2.00 kx interface detail; (**c**,**f**,**i**,**m**) 5.00–25.0 kx nanoparticle distribution.

To validate the proposed reinforcement mechanisms and examine nanoparticle distribution, fracture surfaces of fatigue-tested specimens (failed at 65% UTS) were analyzed using scanning electron microscopy (SEM). Figure 4 presents representative micrographs of all four composite formulations.

The fractographic observations provide direct microstructural evidence supporting the mechanical test results. The baseline composite (C41K41) exhibits clean fiber surfaces and extensive pull-out (Figure 4a–c), confirming weak interfacial bonding as the primary cause of poor fatigue resistance [19,22]. Moreover, graphene-only modification (C43K43) shows matrix remnants on fiber surfaces (Figure 4d–f), demonstrating that nGr enhances fiber–matrix adhesion, delaying debonding initiation under cyclic loading [21,24].

The sub-optimal hybrid (C401K401) reveals partial matrix coverage alongside visible nanoparticle agglomerates (Figure 4g–j, circled). These agglomerates act as stress concentrators that initiate micro-cracks under fatigue loading, explaining why this formulation underperforms despite containing both nanoparticle types [37]. Finally, the optimal hybrid (C407K407) displays exceptional characteristics (Figure 4k–m): complete matrix coverage on all fibers, extensive matrix ductility, and perfectly dispersed nanoparticles (Figure 4m, arrows). The SiO_2_ nanoparticles are uniformly distributed without agglomeration, while GO sheets are well-exfoliated and integrated into the matrix. This ideal multi-scale architecture enables:

- Crack deflection by SiO_2_ nanoparticles, forcing tortuous crack paths (Figure 4l).
- Interfacial strengthening by GO, preventing debonding
- Synergistic toughening where both mechanisms operate simultaneously [25].

These observations directly confirm that the superior fatigue performance of C407K407 (85% higher stress, >63,000 cycles) stems from optimal nanoparticle dispersion and the resulting multi-scale reinforcement mechanisms proposed in Section 3.3.

## 4. Conclusions

This experimental study successfully investigated the high-cycle fatigue behavior of carbon–Kevlar/epoxy hybrid composites modified with nano-silica (nSiO_2_) and nano-graphene (nGr). The key conclusions are as follows:

1. The incorporation of nanoparticles significantly enhances both the static tensile strength and the fatigue life of carbon–Kevlar/epoxy composites. Through systematic Taguchi DOE with 16 formulations, optimal nanoparticle combinations were identified, with UTS improving from 211 MPa (baseline) to 390 MPa (optimal hybrid)—an 85% increase.
2. A synergistic effect is observed when nSiO_2_ and nGr are used in combination. The optimal formulation identified was 1.2 wt.% nSiO_2_ with 0.75 wt.% nGr (C407K407). This hybrid nanocomposite demonstrated exceptional fatigue performance, sustaining 253 MPa at 65% UTS for over 63,000 cycles, compared to the baseline composite which failed at 137 MPa after approximately 54,000 cycles. This represents an ability to operate at an 85% higher stress level for a comparable lifespan.
3. Fractographic examination provided direct visualization of the reinforcement mechanisms:
  - Baseline composite (C41K41) exhibited clean fiber surfaces and extensive pull-out, confirming weak interfacial bonding as the primary cause of poor fatigue resistance.
  - Graphene-only modification (C43K43) showed matrix remnants on fiber surfaces, demonstrating that nGr enhances fiber–matrix adhesion.
  - The sub-optimal hybrid (C401K401) revealed partial matrix coverage alongside visible nanoparticle agglomerates, which act as stress concentrators under cyclic loading explaining why this formulation underperforms despite containing both nanoparticle types.
  - The optimal hybrid (C407K407) displayed complete matrix coverage on all fibers, extensive matrix ductility, and perfectly dispersed nanoparticles with no agglomeration. SiO_2_ nanoparticles are uniformly distributed to provide crack deflection, while GO sheets are well-exfoliated to strengthen interfaces.
4. The enhancement is attributed to complementary mechanisms directly observed via SEM: nSiO_2_ provides matrix toughening and crack deflection, while nGr strengthens the fiber–matrix interface and acts as a crack-bridging agent. The optimal formulation uniquely exhibits both mechanisms operating simultaneously, creating a true multi-scale toughening architecture that maximizes fatigue resistance.
5. A key finding is that static strength alone does not predict fatigue performance. The sub-optimal hybrid (C401K401) achieved higher UTS (378 MPa) than the graphene-only formulation (352 MPa) but showed inferior fatigue life due to nanoparticle agglomeration. Under cyclic loading, agglomerates act as stress concentrators that initiate premature failure—a phenomenon only revealed through combined mechanical testing and fractographic analysis.

This research confirms that strategic multi-scale reinforcement using hybrid nanoparticles is a highly effective and practical method for developing next-generation fatigue-resistant composite materials. However, to fully realize such improvements in practical applications, it is necessary to conduct extensive experiments under realistic and combined loading conditions. Future work will focus on the effects of loading parameters including frequency and stress ratio, and ultimately proportional and non-proportional multiaxial loadings.

## Figures and Tables

**Table 1:** Properties of the epoxy resin system.

Property	Standard	Value (R630)	Value (H630)
Appearance	-	Transparent liquid	Slightly yellow liquid
Viscosity at 25 °C	ISO 12058-1 [31]	1500 cP	30 cP
Density at 25 °C (gr/cm^3^)	ISO 1675 [32]	1.15	1.05
Mix ratio (phr)	-	100	30
Flexural strength (MPa)	ISO 178 [33]	110–117	-
Tensile strength (MPa)	ISO 527 [34]	86.1	68.9
Glass transition temp (°C)	ISO 11359 [35]	118	80

**Table 2:** Properties of reinforcing fibers. Reprinted from Ref. [36].

Property	Unit	CF	KF
Tensile strength	MPa	2500	2800
Tensile modulus	GPa	250	110
Density	kg/m^3^	1.9	1.5
Thickness	mm	0.1	0.37
Areal weight	g/m^2^	107	220

**Table 3:** Composite formulations and sample coding.

Sample Code	nSiO_2_ (wt.%)	nGr (wt.%)	Layup Sequence
C41K41	0	0	[CF/CF/KF/KF/KF/KF/CF/CF]
C43K43	0	0.5	
C401K401	1	0.3	
C407K407	1.2	0.75	

**Table 4:** Taguchi DOE control factors and levels.

Factor	Level 1	Level 2	Level 3	Level 4
nSiO_2_ (wt.%)	0	0.75	1.0	1.2
nGr (wt.%)	0	0.3	0.5	0.75

**Table 5:** Laboratory tensile strength for multi-scale epoxy–carbon fiber–Kevlar composites modified with nanoparticles.

Test Number	Sample Code	Tensile Force (N)	Tensile Strength (MPa)	Average Tensile Strength (MPa)	Thickness (mm)	Width (mm)	Elastic Modulus (GPa)	Average Elastic Modulus (GPa)	Graphene Nanoparticles (%)	Silica Nanoparticles (%)
1	C41K41	16,127	167	211	3	32	27.3	35.3	0	0
		16,712	217		3.5	22	39.6			
		21,300	250		3.7	23	39			
2	C42K42	27,547	254	245	3.1	35	35.3	29.3	0.3	0
		25,011	235		3	35.5	34.8			
		32,700	245		3.8	35	18			
3	C43K43	39,290	362	352	3.1	35	34.8	41.6	0.5	0
		34,200	283		3.5	34.5	40			
		44,900	413		3.1	35	49			
4	C44K44	12,508	219	258	3	19	41.2	35.9	0.75	0
		21,524	368		3	19.5	39.3			
		10,966	187		3	18.5	26.8			
5	C45K45	44,407	328	271	3.7	36.5	36.4	32.8	0	0.75
		14,287	107		3.5	38	26.9			
		23,980	380		3	21	35.2			
6	C46K46	16,600	225	259	3.5	21	33.9	34.5	0.3	0.75
		18,000	267		3.2	21	37.8			
		21,100	287		3.5	21	31.9			
7	C47K47	32,034	228	216	3.6	39	47.4	37.2	0.5	0.75
		35,048	230		3.9	39	40.7			
		29,180	192		3.9	39	23.6			
8	C48K48	32,956	308	305	3.1	34.5	30.9	41	0.75	0.75
		21,800	311		3.5	20	46.9			
		23,100	296		3.9	20	45.3			
9	C49K49	19,200	290	261	3.3	20	31.7	35.3	0	1
		17,200	245		3.5	20	29.2			
		15,500	250		3.1	20	45.1			
10	C401K401	21,800	281	378	3.1	25	29.4	28.3	0.3	1
		34,800	449		3.1	25	28.8			
		25,500	404		3	21	26.9			
11	C402K402	19,970	333	291	3	20	39	31	0.5	1
		16,790	280		3	20	22			
		15,670	260		3	20	31			
12	C403K403	19,410	231	257	4.3	19.5	31	33	0.75	1
		19,450	285		3.5	19.5	37			
		19,960	260		3.9	19.7	33			
13	C404K404	26,849	317	272	3.9	34.5	41.7	36.1	0	1.2
		26,187	265		3.9	34	38.8			
		32,000	234		3.9	35	27.8			
14	C405K405	18,580	317	290	3	19.5	35	36	0.3	1.2
		15,550	266x		3	19.5	37			
		16,035	287		3.1	18	36			
15	C406K406	22,720	315	313	3.6	20	31	36	0.5	1.2
		22,000	305		3.6	20	38			
		23,055	320		3.6	20	39			
16	C407K407	32,590	379	390	3.5	25.3	45	45	0.75	1.2

## Data Availability

The raw data supporting the conclusions of this article will be made available by the authors on request.

## Appendix A

**Table A1:** Axial fatigue testing results of the four selected nanoparticle-reinforced multi-scale composites in this study.

Test No.	Sample Code	Loading Coefficient (Metric: UTS)	Maximum Loading (MPa)	Number of Cycles to Failure
1	C407K407	65%	253	63,365
2		65%	253	66,716
3		65%	253	61,782
4		65%	253	65,438
5		70%	273	40,135
6		70%	273	*
7		70%	273	38,493
8		70%	273	40,562
9		75%	290	29,401
10		75%	290	27,943
11		75%	290	31,440
12		75%	290	30,567
13	C401K401	65%	244	27,405
14		65%	244	30,173
15		65%	244	29,422
16		65%	244	31,005
17		70%	262	15,240
18		70%	262	15,530
19		70%	262	14,879
20		70%	262	17,150
21		75%	282	12,001
22		75%	282	10,651
23		75%	282	9807
24		75%	282	10,379
25	C43K43	65%	228	40,830
26		65%	228	50,567
27		65%	228	47,902
28		65%	228	48,971
29		70%	246	30,370
30		70%	246	31,552
31		70%	246	28,971
32		70%	246	29,805
33		75%	262	23,025
34		75%	262	22,148
35		75%	262	25,061
36		75%	262	*
37	C41K41	65%	137	56,504
38		65%	137	51,431
39		65%	137	54,831
40		65%	137	52,718
41		70%	147	38,761
42		70%	147	42,722
43		70%	147	40,028
44		70%	147	39,617
45		75%	158	27,524
46		75%	158	29,333
47		75%	158	25,834
48		75%	158	28,012

* Specimen failed prematurely due to grip-related issues; data excluded from analysis.
